# Prognostic Significance of Microvessel Density Determining by Endoglin in Stage II Rectal Carcinoma: A Retrospective Analysis

**DOI:** 10.1155/2015/504179

**Published:** 2015-05-21

**Authors:** Zeljko Martinovic, Drazen Kovac, Mia Martinovic

**Affiliations:** ^1^Department of Surgery, Croatian Hospital “Dr. fra Mato Nikolic”, 72 276 Nova Bila, Bosnia and Herzegovina; ^2^Department of Pathology, School of Medicine, University of Rijeka, 51 000 Rijeka, Croatia

## Abstract

*Background*. The role of endoglin in the Dukes B rectal cancer is still unexplored. The aim of this study was to examine the expression of endoglin (CD105) in resected rectal cancer and to evaluate the relationship between microvessels density (MVD), clinicopathological factors, and survival rates. *Methods*. The study included 95 primary rectal adenocarcinomas, corresponding to 67 adjacent and 73 distant normal mucosa specimens from surgical resection samples. Tumor specimens were paraffin-embedded and immunohistochemical staining for the CD105 endothelial antigen was performed to count CD105-MVD. For exact measurement of the CD105-MVD used a computer-integrated system Alphelys Spot Browser 2 was used. *Results*. The intratumoral CD105-MVD was significantly higher compared with corresponding adjacent mucosa (*P* < 0.0001) and distant mucosa specimens (*P* < 0.0001). There was no significant difference in the CD105-MVD according to patients age, gender, tumor location, grade of differentiation, histological type, depth of tumor invasion, and tumor size. The overall survival rate was significantly higher in the low CD105-MVD group of patients than in the high CD105-MVD group of patients (log-rank test, *P* = 0.0406). *Conclusion*. CD105-assessed MVD could help to identify patients with possibility of poor survival in the group of stage II RC.

## 1. Introduction

According to International Agency for Research on Cancer (IARC), colorectal cancer is the third most common cancer and the fourth leading cause of cancer death worldwide [1]. In the European Union (EU), colorectal cancer is the third most common cancer site and the second most common cause of death from cancer [2]. In the Federation of Bosnia and Herzegovina, rectal cancer is the third most common form of cancer in men (16,5%) and the eighth most common in women (10,0%) [3]. Despite significant improvements in the treatment of primary rectal cancer achieved during the last two decades, the long-term outcome of affected patients is still poor [4, 5]. The type of therapy for rectal cancer depends on the tumor location and stage. The pelvic localization of the rectum limits the possibility of radical surgical resection which increases the risk of poor overall prognosis [6]. The group of stage II rectal cancers (pT3-pT4, N0, and M0) includes tumors with different pathological characteristics and variable clinical behavior whose outcomes differ greatly. In patients with stage II supplemental risk estimation is crucial for treatment [7]. The traditional method of stratifying patients with rectal cancer cannot fully predict individual clinical outcome in this group [8].

Tumor growth and its spread to adjacent tissues depend on its ability to stimulate angiogenesis. Angiogenesis consists of formation of new blood vessels from preexisting vasculature [9]. The studies have shown that the angiogenic potential of a tumor may be inferred from its vascularity measured in histological section [10]. The count of blood microvessels of the tumor, as shown in microvessel density, has been recognized as an indicator of malignant potential of the tumors and provides the ability to predict tumors recurrence and survival rate. Many studies have concluded that MVD is inversely related to survival in colorectal cancer [11].

Tumors promote angiogenesis by secreting proangiogenic factors. The transforming growth factor-*β* (TGF-*β*) is a large family of cytokines that play a key role in tumor angiogenesis. Endoglin (CD105) is a type III TGF-*β* coreceptor and it is overexpressed on tumor neovasculature [12]. Endoglin has been suggested to be the most suitable marker available to quantify tumor angiogenesis [13]. In studies, increased CD105 expression as determined by immunohistochemical staining was associated with shorter overall survival rates, but these findings have not always been confirmed [14]. Our study aimed to examine immunohistochemical expression of CD105 in stage II rectal carcinomas and to investigate a correlation between CD105-assessed MVD and clinicopathological variables and to analyze prognostic value of MVD in the overall survival.

## 2. Methods

### 2.1. Patients and Specimens

We studied 95 cases of primary rectal adenocarcinomas in stage II (T3-T4, N0, and M0) treated by complete surgical resection (R0) in a 5-year period at Clinic for Surgery, Clinical Hospital Center Rijeka, Croatia, from January 2002 to December 2006. The study included 95 primary rectal adenocarcinomas, 95 adjacent normal mucosa specimens, and 95 distant normal mucosa specimens from surgical resection samples. The adjacent and distant normal mucosa corresponding to the primary tumor from the same patients were taken from the margin of near and distant surgical resection. Tissue samples included in this study were retrieved from the archives of the Institute of Pathology School of Medicine of Rijeka, Croatia. The exclusion criteria were a synchronous tumor or tumors in another localisation in anamnesis, emergency surgery, preoperative radiotherapy or chemotherapy, perforation of bowel, and incomplete clinical data. The study was approved by the University of Rijeka Ethics Committee and patients signed informed consent.

All of the patients underwent radical low anterior or abdominoperineal rectum resection. All patients had confirmed rectal adenocarcinomas by histopathology and were staged according to the 7th edition of the American Joint Committee on Cancer (AJCC) Staging Manual [15]. The histological grading was classified according to the WHO (World Health Organization) classification [16]. The mean duration of follow-up was 54.7 ± 23.1 months (median duration, 60,0 months) after the operation for rectal cancer (RC). Survival data and cause of death of those who died during follow-up period were obtained from the Croatian Cancer Registry. Patient and tumour characteristic are presented in Table 1.

### 2.2. Immunohistochemistry

Immunohistochemical analysis was performed on formalin-fixed paraffin-embedded section. All tissue samples from RC and adjacent and distant mucosa were fixed in 10% buffered formalin and embedded in paraffin. We prepared 4 *μ*m thick serial sections which were deparaffinized in xylene, rehydrated in graded ethanol, and washed with phosphate-buffered saline. Endogenous peroxidase was inhibited with 3% hydrogen peroxide. Tissue sections were incubated for 30 minutes with the anti-CD105 primary monoclonal antibody (mouse anti-human, clone SN6h, Dako Corporation, Denmark) at a 1 : 10 dilution. Primary antibody binding site was visualized using a secondary antibody detection kit (Envision + kit; Dako, Denmark).

The staining was visualized with diaminobenzidine (DAB). Tissue sections were counterstained with hematoxylin. Brown staining for CD105 was considered positive. Distant normal mucosa free of tumor was used as positive controls and the primary antibody was replaced with phosphate-buffered saline solution for negative controls.

### 2.3. Evaluation of Staining and of MVD by Computerized Image Analysis

All slides stained with anti-CD105 were viewed and analyzed with Alphelys Spot Browser 2 integrated system, using a software controlled (Alphelys Spot Browser 2.4.4., France) stage positioning Nikon Eclipse 50i microscope mounted 1360 × 1024 resolution Microvision CFW-1310C digital camera. The slides were scanned at ×20 magnification to identify “hot spots” (areas with the highest microvessel concentration) for the slides and then at ×200 magnification to create images for quantification scoring positive cells and MVD. Positive cells were counted in the tumor and adjacent and distant normal mucosa and presented as percentage of positive cells and MVD as number of microvessels in the histological field according to Weidner et al. [17]. The regions with the most intensive vascularization (“hot spots”) were defined by scanning the entire tumor section at low magnification with a selection of four fields. The area of this histological field was 0,612 mm^2^. “Hot spots” were identified by two independent observers at ×20 magnification. Semiquantitative expression levels of CD105 were classified according to the following criteria: cases with <1% positive cells (negative staining), 1–25% positive cells (weak staining), 26–50% positive cells (moderate staining), and >50% positive cells (strong staining) as described by Dassoulas et al. [18].

### 2.4. Statistical Analysis

Statistical analysis was performed using MedCalc version 14.8.1 (MedCalc Software bvba, Mariakerke, Belgium). Descriptive statistics and 95% confidence intervals were calculated to describe data. The distribution of data was tested for normality using the Smirnov-Kolmogorov test. McNemar's test was applied to examine the significance of the differences in CD105 expression in tumor, adjacent mucosa, and distant normal mucosa. The Mann-Whitney *U* test and Kruskal-Wallis tests were used to compare MVD among the clinicopathological variables. The receiver operating curve (ROC) approach was used to determine best-fitting cut-off for the CD105 expression and MVD in terms of the survival analysis [19]. Survival analysis was estimated by the Kaplan-Meier method and compared by the log-rank test. Prognostic factors of survival were identified by the use of the Cox proportional hazard regression. Differences at *P* < 0.05 were considered significant.

## 3. Results

### 3.1. Patient Sample Classification

We assessed paraffin-embedded specimens from tumors from 95 patients resected for RC. Clinicopathological characteristics of patients are summarized in Table 2. The median age at diagnosis was 69 years (range 15 to 85 years), 49 patients (51.6%) were ≤69 years of age, and 46 patients (48.4%) were >69 years old. Sixty-one (64.2%) were males and 34 (35.8%) were females. In 23 patients (24.2%), the tumor was located in the upper rectum, in 52 (54.7%) in the middle rectum and in 20 (21.1%) in the low rectum. According to grade of differentiation, 55 patients (57.9%) were G1 (well differentiated), 34 (35.8%) G2 (moderately differentiated), and 6 (6.3%) G3 (poorly differentiated). According to depth of tumor invasion, 37 patients (38.9%) were T3, 42 (44.2%) T4a, and 16 (16.9%) T4b. Eighty-two (86.3%) tumors were classified as adenocarcinomas and 13 (13.7%) as adenocarcinomas with mucinous features. Median tumor size was 3.8 cm (range, 1,3 to 12,0 cm). The median patients follow-up was 60 months (range, 1.0 to 109.0 months). Of the 95 patients, 15 patients developed recurrent disease and 29 died of RC in the 5-year follow-up period.

### 3.2. CD105 Expression in RC Samples

**Figure 1:**
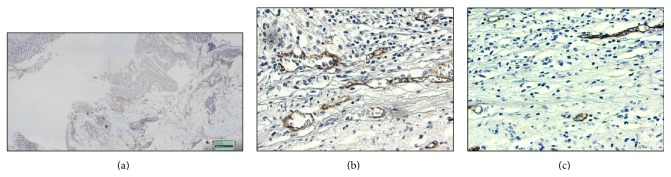
Immunohistochemical endoglin expression in rectal carcinoma (a), magnification ×20. High endoglin expression (b) and low endoglin expression (c). Magnification ×200.

**Figure 2:**
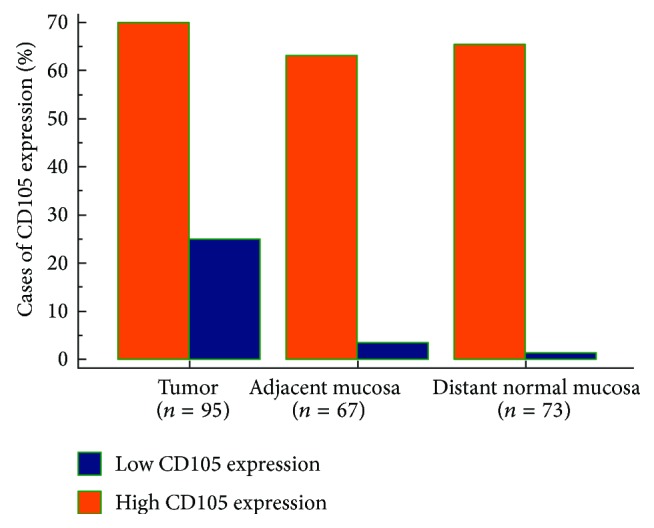
The count of the CD105 expression in the tumour, adjacent normal mucosa, and distant normal mucosa. The count of the CD105 expression was significantly higher in the tumour compared with the adjacent normal mucosa (*P* = 0.0015) and the distant normal mucosa (*P* < 0.001).

The CD105 (endoglin) expression was analyzed by immunohistochemistry in the tumors, adjacent normal mucosa, and distant normal mucosa. The CD105 expression was detected on cell membrane of the endothelial cells in all the sites. Examples of low and high CD105 expression in the tumors are shown in Figure 1. Endoglin staining was observed in 93 of 95 tumours (97.9%). Most specimens (71.6%) had weak to moderate CD105 staining intensity, while 26.3% of specimens had strong staining intensity. Strong CD105 staining was found in 4 (5.9%) samples of adjacent normal mucosa and in only one (1.3%) sample of distant normal mucosa. Using a cut-off value of ≤48,8%, tumors were divided into two groups: low CD105 expression and high CD105 expression. The count of the CD105 expression in the tumors, adjacent mucosa, and distant normal mucosa is shown in Figure 2. McNemar's test was performed. The CD105 expression in the tumor was significantly higher compared with the adjacent mucosa (*P* = 0.001) and the distant normal mucosa (*P* < 0.001; Figure 2). There was no significant difference in CD105 expression in the adjacent mucosa and distant mucosa (*P* = 0.375).

### 3.3. Microvessel Density

Significant correlation between CD105 expression and MVD in tumors was determined by Spearman's coefficient of rank (rho = 0.602, *P* < 0.0001, 95% CI 0.456 to 0.717). MVD were analyzed in tumors, adjacent mucosa, and distant mucosa. Median CD105-assessed MVD in tumors was 174.47 vessels/mm^2^ (95% CI 151.00–205.29), in adjacent mucosa 88.24 vessels/mm^2^ (95% CI 55.46–103.80), and in distant mucosa 58.82 vessels/mm^2^ (95% CI 51.42–82.56). The MVD was significantly higher in the tumor samples compared with adjacent mucosa (*P* < 0.0001) and the distant mucosa (*P* < 0.0001). There was no significant difference in the MVD according to patients age, gender, tumor location, grade of differentiation, histological tumor type, depth of tumor invasion, and tumor size (Table 2).

### 3.4. Univariate Survival Analysis

**Figure 3:**
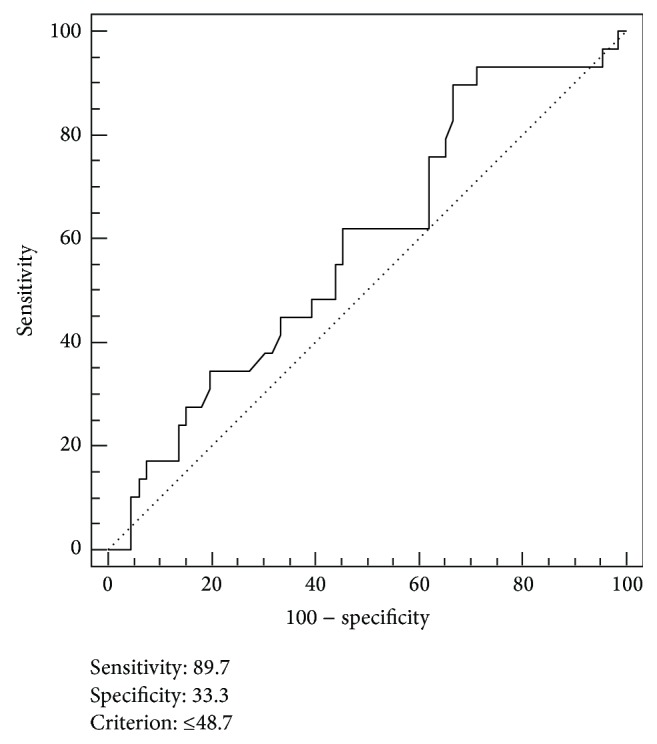
Receiver operating characteristic (ROC) curve analysis for optimal cut-off point of CD105 expression. Using a cut-off value of ≤48,8%, tumors were divided into two groups: low CD105 expression and high CD105 expression. ROC analysis indicated an optimal cut-off of 48,7% in prognostic survival in stage II rectal cancer. Area under the ROC curve (AUC): 0.586, *P* = 0.171.

The Kaplan-Meier method and long-rang test were performed. There were significant differences in survival rates in the groups of patients with ≤ and >69 years old (*P* = 0.0156; Table 3). The group of patients with ≤69 years of age had higher survival rate than patients >69 years of age. High CD105 expression was identified in 24 (25,3%) of 95 cases and low or negative expression in 71 cases (74,7%). CD105 expression showed a significant effect on patient survival (Figure 3). Patients with high CD105 expression had significantly longer overall survival time, compared to CD105 low expression group (log-rank test, *P* = 0.035).

**Figure 4:**
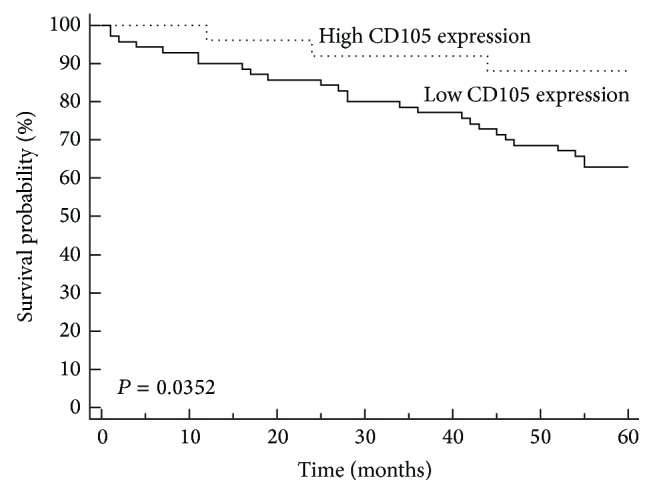
Kaplan-Meier analysis of overall survival for stage II rectal cancer patients according to low and high CD105 expression. CD105 expression shows significant correlation with survival of patients with stage II rectal carcinoma (*P* = 0.035).

**Figure 5:**
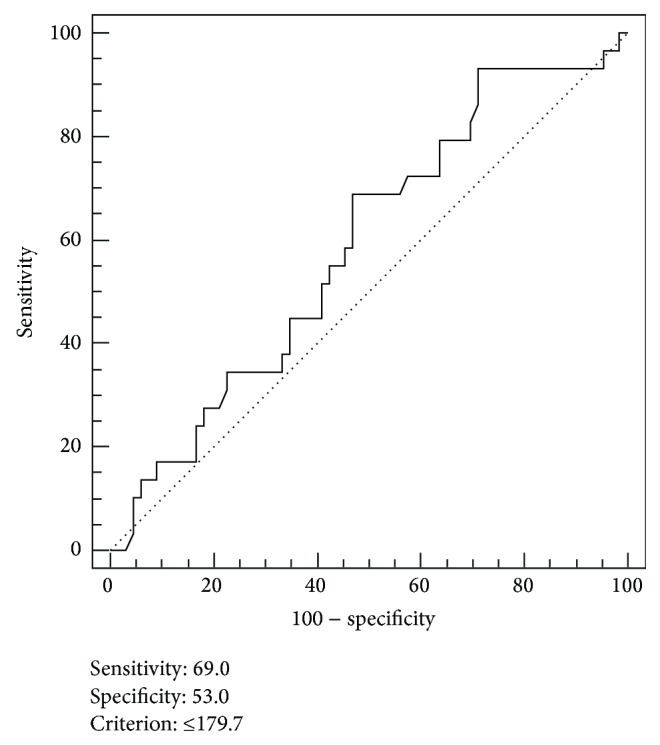
Receiver operating characteristic (ROC) curve analysis for optimal cut-off point of CD105-MVD. According to the ROC curve analysis the hypervascularized tumours were discriminated from the hypovascularized tumours. ROC analysis indicated an optimal cut-off of 179.7 microvessel/mm^2^ in prognostic survival in stage II rectal cancer (Figure 6). Area under the ROC curve (AUC): 0.587, *P* = 0.165.

The cut-off value for determining high and low MVD was performed by the receiver operating characteristic (ROC) curve analysis. The cut-off value was ≤179.7 microvessel/mm^2^ (sensitivity 69.0%, specificity 53.0%) (Figure 4). In a Kaplan-Meier survival estimate, the overall survival (OS) rate was significantly higher in the high MVD group of patients than the low MVD group of patients (Figure 5, log-rank test, *P* = 0.040).

### 3.5. Multiple Cox Regression Analysis

The prognostic variables were determined by Cox proportional hazard regression analysis. The full model containing all variables was statistically significant (*P* < 0.05), indicating that this model was able to distinguish between survival and nonsurvival. As shown in Table 4, three variables significantly affected the model, age, CD105 expression, and CD105-assessed MVD. “Backward” analysis was performed. The result showed that age (OR = 1.05, *P* = 0.0110), CD105 expression (OR = 1.03, *P* = 0.0485), and CD105-assessed MVD (OR = 0.31, *P* = 0.0370) were the independent prognostic factors for OS (Table 4).

## 4. Discussion

Stage II RC is defined by the presence of penetration through the muscularis propria and the absence of metastasis to either regional lymph nodes or distant sites [19]. However, death from RC of stage II continues to occur in approximately 20% of patients [20, 21]. Identifying high-risk patients with stage II RC is important because it my help to identify patients and additional risk for whom surgery alone may not be curative treatment. Endoglin is a proliferation-associated antigen on endothelial cells and essential for angiogenesis. It has been reported that expression of the endoglin in tumor endothelium may be a prognostic indicator of the outcome for various humans tumors including and colorectal cancer (CRC) [22]. These findings have not been confirmed by all researchers.

In the present study we analyzed the relationship between the CD105 expressions in RC, adjacent normal mucosa, and distant normal mucosa. In our cohort CD105 positive immunostaining was found in 97.9% of all rectal tumour specimens tested, with most samples having weak to moderate staining intensity (71.6%). We showed that the CD105 expression levels significantly increase in RC from the distant and adjacent mucosa to the primary tumor (Figure 2). Endoglin was expressed at low level in endothelial cells in normal mucosa of the rectum and strongly expressed in vascular endothelial cells in tumor vessels. Our study has shown that CD105 is upregulated in RC tissue in comparison with normal mucosa of rectum. The intensity of staining for endoglin indicates that endoglin is a powerful marker of neovascularization in RC. This pattern of expression of the endoglin shows its role as proangiogenic component in tumor endothelial cells.

Previous studies have demonstrated that increased endoglin expression assessed immunohistochemically correlates with the decreased survival period. Svagzdys et al. and Dassoulas et al. showed a clear association of cancer-specific OS with high CD105 expression [18, 23]. We also analyzed the relationship between the CD105 expression and patients survival. Our results showed that there was significant correlation between low CD105 expression and short survival in the cohort of our RC patients. Patients with high CD105 expression had a good prognosis.

Microvessel density assessment is the most commonly used technique to quantify intratumoral angiogenesis in cancer. In the present study, we assessed MVD with CD105 marker in RC tissue and adjacent and distant normal mucosa. Endoglin microvessel immunostaining was consistently present in all the cases studied. Consistent with previous studies, we found a significant increase of MVD in RC compared with their corresponding adjacent and distant normal mucosa [24, 25]. These results support the role of CD105 as an optimal marker of proliferation of endothelial cells and its potential as prognostic factor [13, 14, 26]. The results of previous studies have shown that the use of CD105-MVD does not correlate with other histopathological parameters in the cohort of RC (Svagzdys et al.) or CRC (Dassoulas et al., Saad et al.) [18, 23, 27]. In our study, we also have not found a statistically significant correlation between CD105-MVD and conventional histopathological parameters in the cohort of stage II RC patients (Table 2).

Survival rates in our cohort stage II RC patients were analyzed according to age, gender, tumor location, grade of differentiation, histology, CD105 expression, and MVD. By univariate analysis, age, MVD, and CD105 expression were found to be significant prognostic factors for OS. In patients older than 69 years, low MVD and low CD105 expression were of high risk, with short median of OS. In our cohort, decreased CD105-MVD was associated with decreased OS. In multivariate analysis (Table 4) by the Cox proportional hazard regression model, age, CD105 expression, and CD105-MVD had significance as independent prognostic factor for OS.

Patients with stage II RC have poor survival despite multimodality treatment [28]. Although angiogenesis affects the outcome of treatments, the importance of angiogenesis as a prognostic factor is still not clearly enough defined. In the studies, there are considerable differences in microvessel counts in tissue of rectal carcinoma. The quantification of microvessel density was made in the majority of studies with classical Weidner's method [17]. In our study, tumor microvessel density was obtained by computerized image analysis.

For colorectal cancer, conflicting results have been reported on the prognostic importance of MVD in various subsets of patients. Due to inconsistent methods of analysis of tumor angiogenesis in various studies, it is difficult to compare the values of MVD obtained in our analysis with the results of other authors. In our analysis, we found higher values of MVD (CD105-MVD, 221.0/mm^2^ on average) in RC tissues in comparison with the results in the study of Svagzdys et al. (CD34-MVD, 193.0/mm^2^ on average), possibly due to the larger surface of the analyzed tumor tissue (0.612/mm^2^ versus 0.576/mm^2^) and the use of different endothelial cell markers [23]. In the present study, the microvessel counts are high and confirm that the rectal carcinoma is strongly dependent on angiogenesis.

Furthermore, significantly lower rates of survival were found in patients with lower MVD than cut-off value obtained by ROC analysis. This is shown by Kaplan-Meier survival curves for MVD variable (Figure 5). Our results suggest that the higher CD105-MVD accompanied by good OS, which is not in accordance with the results of most of the authors that a high MVD accompanied with poor OS [23]. Prall et al. have reported higher survival rate in tumors with high MVD (factor VIII immunostaining, 0.74 mm^2^, median as cut-off) which is consistent with our results [29]. According to the results of Uribarrena et al., patients with stages I and II colorectal carcinomas with higher vascularized tumor area had a significant association with a better outcome, but no significant relationship was observed between MVD and tumor recurrence and death [30]. Some studies demonstrated that high MVD counts determined using CD105 were strongly associated with a poor survival rate (Li et al., median as cut-off) and high risk of metastatic disease (Saad et al., Romani et al., median as cut-off) [27, 31, 32].

However, the results of different studies linking lower MVD in tumors with poor survival and in various other solid tumors [33]. Endoglin is an auxiliary membrane receptor for transforming growth factor-beta (TGF-*β*) that modulates TGF-*β* signaling [33]. Recently, endoglin has been identified as a key regulator of tumor cells proliferation, migration, and invasion [24, 33]. Craft et al. showed that endoglin expression was lost during prostate cancer cell progression and that it led to increased cell invasion and migration [34]. It has been suggested that endoglin deficiency results in angiogenic adaptation, weakens the endothelial barrier, and increases metastatic spread, and may be associated with cancer progression [35].

## 5. Conclusion

In conclusion, this study showed that the CD105 expression and CD105-MVD are useful markers for identifying patients with an aggressive form of stage II RC. CD105-assessed MVD could help to identify patients with possibility of poor survival in the group of stage II RC.

## Figures and Tables

**Figure 6:**
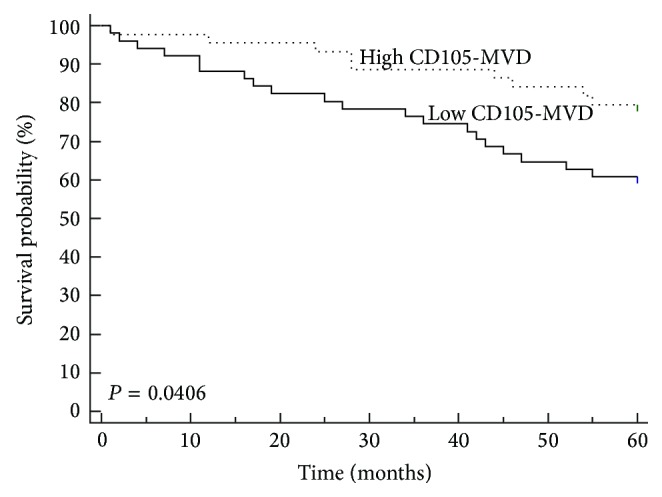
Kaplan-Meier curve for patients with stage II rectal cancer according to microvessel density (MVD) by CD105. CD105 microvessel density correlates significantly with survival of patients with stage II rectal cancer (179.7 microvessels/mm^2^, as the cut-off).

**Table 1:** Clinical and pathologic characteristics of the rectal cancer samples.

Characteristics	Number of patients
Total number	95
Age, median 69 years	
≤69	49
>69	46
Gender	
Male	61
Female	34
Surgery	
Low anterior resection	76
Abdominoperineal resection	19
Tumor location	
Upper rectum	23
Middle rectum	52
Low rectum	20
Grade of differentiation	
G1	55
G2	34
G3	6
Histologic type	
Adenocarcinoma	82
Adenocarcinoma with mucinous features	13
Depth of tumor invasion	
T3	37
T4a	42
T4b	16
Tumor size	
≤4 cm	64
>4 cm	31

**Table 2:** CD105-MVD and clinicopathological factors in patients with rectal cancer.

Variable	Patients *n* (%)	CD105-MVD median (95% CI)	*P* value
Age, median 69 years			0.268^∗^
≤69	49 (51.6)	192.8 (152.6–296.1)	
>69	46 (48.4)	164.2 (125.8–192.2)	
Gender			0.499^∗^
Male	61 (64.2)	171.5 (124.7–206.0)	
Female	34 (35.8)	184.6 (159.4–247.3)	
Tumor location			0.529^∗∗^
Upper rectum	23 (24.2)	200.9 (165.2–285.8)	
Middle rectum	52 (54.7)	178.9 (136.2–240.5)	
Low rectum	20 (21.1)	125.8 (106.2–194.9)	
Grade of differentiation			0.245^∗∗^
G1	55 (57.9)	171.5 (127.0–192.3)	
G2	34 (35.8)	191.7 (124.1–289.2)	
G3	6 (6.3)	267.1 (105.6–578.2)	
Histologic type			0.160^∗^
Adenocarcinoma	82 (86.3)	173.2 (137.1–193.4)	
Adenocarcinoma with mucinous features	13 (13.7)	235.2 (127.3–423.8)	
Depth of tumor invasion			0.202^∗∗^
T3	37 (38.9)	173.2 (120.1–189.2)	
T4a	42 (44.2)	205.0 (151.1–315.0)	
T4b	16 (16.9)	151.1 (95.4–292.7)	
Tumor size			0.775^∗^
≤4 cm	64 (67.4)	175.6 (131.4–197.8)	
>4 cm	31 (32.6)	196.1 (142.7–244.7)	

^∗^Mann-Whitney *U* test; ^∗∗^Kruskal-Wallis test.

**Table 3:** Overall survival rates and univariate analysis of patients with stage II rectal cancer.

Variable	Number of patients	Overall survival rates	Log-rank test
Age			**0.015**
≤69	49	79.6	
>69	46	58.7	
Gender			0.805
Male	61	70.5	
Female	34	67.6	
Tumor location			0.446
Upper rectum	23	69.6	
Middle rectum	52	65.4	
Low rectum	20	80.0	
Grade of differentiation			0.721
G1	55	72.7	
G2	34	64.7	
G3	6	66.7	
Histologic type			0.581
Adenocarcinoma	82	68.3	
Adenocarcinoma with mucinous features	13	76.9	
CD105 expression			**0.035**
High	24	87.5	
Low	71	63.4	
CD105-MVD			**0.040**
High	44	79.5	
Low	51	60.8	

**Table 4:** The multivariate Cox proportional hazard regression analysis for survival in stage II rectal cancer patients (method “*backward*”).

Covariate	*P*	OR	(95% CI)
Age (≤69 versus >69)	0.0110	1.0572	1.0130–1.1032
CD105 expression (low versus high)	0.0485	1.0373	1.0004–1.0754
CD105-MVD (low versus high)	0.0370	0.3156	0.1074–0.9275

Overall model fit *χ* ^2^ = 20.755, *P* = 0.0004.

MVD: microvessel density.

OR: odds ratio.

95% CI: confidence interval.
